# 2-Pentadecyl-2-Oxazoline, the Oxazoline of Pea, Modulates Carrageenan-Induced Acute Inflammation

**DOI:** 10.3389/fphar.2017.00308

**Published:** 2017-05-30

**Authors:** Stefania Petrosino, Michela Campolo, Daniela Impellizzeri, Irene Paterniti, Marco Allarà, Enrico Gugliandolo, Ramona D’Amico, Rosalba Siracusa, Marika Cordaro, Emanuela Esposito, Vincenzo Di Marzo, Salvatore Cuzzocrea

**Affiliations:** ^1^Endocannabinoid Research Group, Institute of Biomolecular Chemistry, Consiglio Nazionale delle RicerchePozzuoli, Italy; ^2^Epitech Group SpASaccolongo, Italy; ^3^Department of Chemical, Biological, Pharmaceutical and Environmental Science, University of MessinaMessina, Italy

**Keywords:** inflammation, PEA, Oxazoline, paw edema, carrageenan 1

## Abstract

*N*-acylethanolamines (NAEs) involve a family of lipid molecules existent in animal and plant, with *N-*palmitoylethanolamide (PEA) that arouses great attention owing to its anti-inflammatory, analgesic and neuroprotective activities. Because PEA is produced on demand and exerts pleiotropic effects, the modulation of specific amidases for NAEs (and in particular NAE-hydrolyzing acid amidase NAAA, which is more selective for PEA) could be a condition to preserve its levels. Here we investigate the effect of 2-Pentadecyl-2-oxazoline (PEA-OXA) the oxazoline of PEA, on human recombinant NAAA *in vitro* and in an established model of Carrageenan (CAR)-induced rat paw inflammation. PEA-OXA dose-dependently significantly inhibited recombinant NAAA and, orally administered to rats (10 mg/kg), limiting histological damage, thermal hyperalgesia and the increase of infiltrating inflammatory cells after CAR injection in the rat right hindpaw, compared to ultramicronized PEA given orally at the same dose (10 mg/kg). These effects were accompanied by elevation of paw PEA levels. Moreover, PEA-OXA markedly reduced neutrophil infiltration and pro-inflammatory cytokine release and prevented CAR-induced IκB-α degradation, nuclear translocation of NF-κB p65, the increase of inducible nitric oxide synthase, cyclooxygenase-2, intercellular adhesion molecule-1, and mast cell activation. Experiments in PPAR-α knockout mice showed that the anti-inflammatory effects of PEA-OXA were not dependent on the presence of PPAR-α receptors. In conclusion, NAAA modulators as PEA-OXA could help to maximize the tissue availability of PEA by increasing its levels and anti-inflammatory effects.

## Introduction

Inflammation is an adaptive response that includes vascular and cellular events which play a key role in evading the injurious stimulus and normalizing the disturbed tissue homeostasis. Acute inflammation begins within seconds to minutes following tissue injury; it is normally a short-lived phenomenon and is accompanied by localized intensification in blood flow, neutrophil infiltration, cytokine release and pain (Medzhitov, 2008; Begum et al., 2015). The production of prostaglandins (PGs), through the metabolism of arachidonic acid by cyclooxygenase (COX), is one of the key pathways involved in the pathogenesis of acute inflammation (Egan et al., 2002). COX-2 is an immediate/early gene whose expression in most tissues is low or absent but is transiently induced by mitogens and cytokines. Whereas COX-2 is generally considered proinflammatory, it also appears to play a role in the resolution of inflammation (Bondesen et al., 2004). Inflammation and tissue injury also lead to the development of hyperalgesia and allodynia. Both peripheral and central processes especially in the spinal cord underlie this phenomenon via the production of nitro-oxidative species (NOX) and (COX)-derived prostaglandins (Cordaro et al., 2016).

Although a number of drugs are available to reduce these damaging events, there is a continuous search for new highly efficacious and safe drugs that can satisfy both safety and high efficacy requirements. Palmitoylethanolamide (PEA), a fatty acid amide belonging to the family of *N*-acylethanolamines (NAEs), is currently considered an important endogenous molecule capable of controlling tissue reactivity and the related inflammatory antalgic phenomena, both at innervated peripheral tissues and at the central nervous system (CNS), in particular at the spinal and supra-spinal levels (Costa et al., 2002; Lo Verme et al., 2005). Furthermore, PEA exerts anti-inflammatory effects also in chronic inflammation models, such as collagen-induced arthritis (Impellizzeri et al., 2013). In both animal models and human pathologies, PEA is efficacious at controlling the neuropathic pain induced by lesions or alterations at either the central or peripheral nervous system level (Darmani et al., 2005; Costa et al., 2008; Starowicz et al., 2013). Several mechanisms could explain the anti-inflammatory and anti-hyperalgesic effects of PEA, in particular the activation of a cell surface cannabinoid CB2-like receptor or the orphan GPR55 receptor, or a nuclear receptor of the peroxisome proliferator-activated receptors (PPARs) family (Farquhar-Smith et al., 2002), and the down-modulation of mast cell (MC) degranulation (ALIA mechanism) (Aloe et al., 1993).

Innate immune cells produce significant amounts of lipid amides (Bisogno et al., 1997) and express two intracellular amidases implicated in lipid amide degradation: fatty-acid amide hydrolase (FAAH) (McKinney and Cravatt, 2005) and *N*-acylethanolamine-hydrolyzing acid amidase (NAAA) (Ueda et al., 2001; Tsuboi et al., 2007). FAAH and NAAA are specific hydrolases which degrade NAEs; NAAA, in particular is more important for the degradation of PEA (Tai et al., 2012); however, also oleoylethanolamide (OEA), stearoylethanolamide (SEA) and, to a lesser extent, anandamide (AEA) are hydrolyzed by NAAA (Tai et al., 2012). NAE catabolic enzymes are currently viewed as potential therapeutic targets whose inhibition may increase tissue levels of PEA (Solorzano et al., 2009; Alhouayek et al., 2015; Petrosino et al., 2015; Ribeiro et al., 2015).

Interest in strategies to increase the endogenous levels of NAEs, in particular PEA, has led to the search for new selective molecules of a regulatory nature which do not cause frank inhibition of FAAH and/or NAAA. The regulatory role of these latter enzymes is intended to modulate the availability of substrates, such as PEA, produced on demand by specific cells (e.g., mast cells, microglia, astrocytes) to exert protective actions (Skaper et al., 2015). In this context, it may well be that NAE catabolic enzymes (in particular NAAA) are designed by nature to modulate substrate availability (Della Valle et al., 2014; Skaper et al., 2015). Curiously, oxazoline derivatives of fatty acids have never been evaluated for their ability to inhibit FAAH and/or NAAA or for their possible inhibitory activity of inflammatory processes (Della Valle et al., 2014).

In particular, the present work aimed to demonstrate that a new PEA derivative, PEA-oxazoline (PEA-OXA), which is present in food sources (Impellizzeri et al., 2016a), is able to inhibit NAAA and to markedly reduce inflammation using carrageenan (CAR)-induced edema in the rat paw that is an established model of acute inflammatory pain frequently used for assessing anti-inflammatory drugs (Winter et al., 1962). A preliminary our study has just demonstrated that PEA-OXA given orally had beneficial effects against CAR-induced paw edema (Impellizzeri et al., 2016a). Moreover, because the mechanism of action of PEA appears to involve, at least in part, PPAR-α, we interrogated PEA-OXA effects in PPAR-α knockout (KO) and wild-type (WT) mice.

## Materials and Methods

### Cell Culture

Human embryonic kidney (HEK)-293 cells stably transfected with human recombinant NAAA (HEK-NAAA cells) were cultured in DMEM supplemented with blasticidin (2 mM), penicillin (400 U/mL), streptomycin (50 mg/mL) and 10% Fetal Bovine Serum (FBS) in an humidified 5% CO_2_ atmosphere at 37°C.

### Assay of NAAA and FAAH Activity

Human embryonic kidney-NAAA cells were suspended and homogenized in Tris-HCl 20 mM (pH 7.4). The assay was carried out as previously described (Petrosino et al., 2015). Briefly, the homogenate was centrifuged at 800 × *g* for 10 min and then at 12000 × *g* for 30 min at 4°C. The 12000 × *g* pellet (membranes) was suspended in PBS (pH 7.4), subjected to two cycles of freezing and thawing as previously suggested to increase the availability of the enzyme in the assay. The membranes (50 mg protein/sample) were allowed to react at 37°C for 30 min with 20 mM [^3^H]-*N*-palmitoylethanolamine (15000 c.p.m./sample) in a solution of citrate/sodium phosphate 50 mM (pH 5.2) and 0.1% Triton X-100, containing the test compound. The reaction was terminated by the addition of chloroform/methanol (1:1 by vol.) and quantification of [^3^H]-ethanolamine was carried out by using Liquid Scintillation Analyzer (TRI-carb 2100TR).

Fatty-acid amide hydrolase activity was measured by incubating the membrane fraction (10000 × *g* pellet, 70 mg protein/sample) of rat brain in Tris-HCl 50 mM (pH 9.0–10.00) at 37 °C for 30 min with *N*-arachidonoyl-[^14^C]-ethanolamine ([^14^C]-AEA, 110 mCi/mmol) properly diluted with unlabelled AEA, containing the test compound. The reaction was terminated by the addition of chloroform/methanol (1:1 by vol.) and quantification of [^14^C]-ethanolamine was carried out by using Liquid Scintillation Analyzer (TRI-carb 2100TR).

### Animals

The study was carried out using Sprague–Dawley male rats (200–230 g, Envigo, RMS Srl Udine, Italy) and mice (4–5 weeks old, 20–22 g) with a targeted disruption of the PPAR-α gene. (PPAR-αKO) and littermate WT controls purchased from Jackson Laboratories (Envigo, RMS Srl Udine, Italy).

Mice homozygous for the Pparat^niJ^Gonz targeted mutation mice are viable, fertile and appear normal in appearance and behavior Exon eight, encoding the ligand-binding domain, was disrupted by the insertion of a 1.14 kb neomycin resistance gene in the opposite transcriptional direction. After electroporation of the targeting construct into J1 ES cells, the ES cells were injected into C57BL/6N blastocysts. This stain was created on a B6, 129S4 background and has been maintained as a homozygote on a 129S4/SvJae background by brother sister matings.

Food and water were available *ad libitum*. University of Messina Review Board for the care of animals approved the study. Animal care was in according with Italian regulations on use of animals for experiment (D.M.116192) as well as with EEC regulations (O.J. of E.C. L 358/1 12/18/1986).

### CAR-Induced Paw Edema

Paw edema was induced by a subplantar injection of CAR (100 μl of a 1% suspension in 0.85% saline for rats and 50 μl for mice). Changes in paw volume were measured as previously described in rats and mice (D’Agostino et al., 2007; Impellizzeri et al., 2016b) using a plethysmometer (Ugo Basile, Varese, Italy) immediately prior to CAR injection, and, thereafter, at hourly intervals for 6 h. Edema was expressed as increase in paw volume (ml) after CAR injection relative to pre-injection value for each rat. Results are reported as paw-volume change (ml).

### Experimental Groups

Rats were divided in several groups with different doses of PEA-OXA:

(i) Carrageenan+ vehicle group: rats were subjected to CAR-induced paw edema and received orally by gavage the vehicle (250 μl based on body weight; carboxymethylcellulose (CMC) 2.5% p/p in water; *N* = 10);
(ii) Carrageenan+ PEA-OXA (10 mg/kg) dissolved in vehicle (carboxymethylcellulose (CMC) 2.5% p/p in water): same as the CAR +vehicle group but PEA-OXA (10 mg/kg, by oral gavage, 250 μl based on body weight) was administered 30 min before CAR (*N* = 10);
(iii) Carrageenan+ PEA-OXA (3 mg/kg) dissolved in vehicle (CMC 2.5% p/p in water): same as the CAR +vehicle group but PEA-OXA (3 mg/kg, by oral gavage, 250 μl based on body weight) was administered 30 min before CAR (*N* = 10);
(iv) Carrageenan+ PEA-OXA (1 mg/kg) dissolved in vehicle (CMC 2.5% p/p in water): same as the CAR +vehicle group but PEA-OXA (1 mg/kg, by oral gavage, 250 μl based on body weight) was administered 30 min before CAR (*N* = 10);
(v) Carrageenan+ ultramicronized PEA (10 mg/kg) dissolved in vehicle (CMC 2.5% p/p in water): same as the CAR +vehicle group but ultramicronized PEA (10 mg/kg, by oral gavage, 250 μl based on body weight) was administered 30 min before CAR (*N* = 10).

The second phase of the study was designed to investigate whether the mechanism of action of PEA-OXA is related to activation of PPAR-α. For this PPAR-α KO and WT mice were used.

Peroxisome proliferator-activated receptor-α KO and WT mice were randomly allocated to the following groups:

(i) Carrageenan*+* vehicle group: PPAR-α KO and WT mice were subjected to CAR-induced paw edema and received orally by gavage the vehicle (250 μl based on body weight; carboxymethylcellulose (CMC) 2.5% p/p in water; *N* = 10 for each group of mice);
(ii) Carrageenan+ PEA-OXA (10 mg/kg) dissolved in vehicle (CMC 2.5% p/p in water): same as the CAR +vehicle group but PEA-OXA (10 mg/kg, by oral gavage, 250 μl based on body weight) was administered 30 min before CAR (*N* = 10 for each group of mice).

The sham-operated group underwent the same identical surgical procedures as the CAR group, except that vehicle or drugs were administered instead of CAR (*N* = 10 for all experimental groups).

### LC-APCI-MS Analysis of PEA and Related Mediators

Liquid chromatography-atmospheric pressure chemical ionization-mass spectrometry (LC-APCI-MS) analyses of AEA, 2-AG, PEA and OEA levels were carried out as previously described (Bisogno et al., 1997; Marsicano et al., 2002). Briefly, plantar paws were homogenized in a solution of chloroform/methanol/Tris-HCl 50 mM pH 7.4 (2:1:1 by vol.) containing 10 pmol of [^2^H]_8_-AEA, and 5 pmol each of [^2^H]_5_-2-AG, [^2^H]_4_-PEA and [^2^H]_2_-OEA as internal deuterated standards. The lipid-containing organic phase was pre-purified by open-bed chromatography on silica gel, and fractions obtained by eluting the column with a solution of chloroform/methanol (90:10 by vol.) were analyzed by LC-APCI-MS by using a Shimadzu HPLC apparatus (LC-10ADVP) coupled to a Shimadzu (LCMS-2020) quadrupole MS via a Shimadzu APCI interface. LC-APCI-MS analyses of AEA, 2-AG, PEA and OEA were carried out in the selected ion monitoring (SIM) mode, using *m/z* values of molecular ions +1 for deuterated and undeuterated compounds, respectively, as follows: 356 and 348 (AEA), 384.35 and 379.35 (2-AG), 304 and 300 (PEA), 328 and 326 (OEA). AEA, 2-AG, PEA and OEA levels were calculated on the basis of their area ratio with the internal deuterated standard signal areas, and their amounts (pmol) were normalized per g or mg of plantar paw.

### Behavioral Analysis in Rats

Behavioral testing was done with the experimenter blinded to the treatment conditions. Hyperalgesic responses to heat were determined as previously described (Hargreaves et al., 1988) at different time points (0, 30 min, 1, 2, 3, 4 and 5 h) with a cut-off latency of 20 sec to prevent tissue damage in non-responsive rats. Briefly, animals were placed in plexiglass chambers. The mobile high intensity projector was set to deliver a thermal stimulus directly to a single hind paw. The withdrawal latency period of injected and normal paws was defined to the nearest 0.1 s with an electronic clock circuit and thermocouple. If the rat failed to respond by 20 sec the test was stopped. Each point represents the delta change (sec) in withdrawal latency (withdrawal latency of contralateral minus withdrawal latency of injected paw) at each time point. Results are expressed as paw withdrawal latency changes (sec).

### Determinations of Cytokine Levels in Rat Paw Exudates

Cytokines TNF-α, IL-1β, IL -6 in the paw exudates were measured by ELISA as described previously (Salvemini et al., 1996).

### Histological Examination of the CAR-Inflamed Hind Paw

Biopsies of the palm of hind paws were taken 6 h following CAR injection. Histology was performed as previously described (Impellizzeri et al., 2016b).

The degree of paw damage was evaluated according on a six-point score: 0 = no inflammation, 1 = mild inflammation, 2 = mild/moderate inflammation, 3 = moderate inflammation, 4 = moderate/severe inflammation and 5 = severe inflammation.

### Myeloperoxidase (MPO) Activity

Myeloperoxidase activity, an index of polymorphonuclear cell accumulation, was determined as previously described in the palm of hind paw tissues (Impellizzeri et al., 2013).

The rate of change in absorbance was measured spectrophotometrically at 650 nm. MPO activity was measured as the quantity of enzyme degrading 1 mM of peroxide min^-1^ at 37°C, and was expressed in units per gram weight of wet tissue.

### Western Blot Analysis for IκB-α, Nuclear Factor-kappaB (NF-κB) p65, COX-2, and Inducible Nitric Oxide Synthase (iNOS)

Cytosolic and nuclear extracts of the palm of hind paws and spinal cord tissues were performed as previously described (Impellizzeri et al., 2013). The levels of IκB-α, COX-2 and iNOS were quantified in the cytosolic fraction, while NF-κB p65 levels were quantified in the nuclear fraction. The filters were blocked with 1 × PBS, 5% (w/v) non-fat dried milk for 40 min at room temperature and subsequently probed with one of the following primary antibodies (all from Santa Cruz Biotechnology) IκB-α (1:1000), NF-kB p65 (1:1000), COX-2 (1:500) or iNOS (1:500) in 1 × PBS, 5% w/v non-fat dried milk, 0.1% Tween-20 at 4°C, overnight. Membranes were incubated with secondary antibody (1:2000, Jackson ImmunoResearch) for 1 h at room temperature.

### Immunohistochemistry for Intercellular Adhesion Molecule 1 (ICAM-1)

Immunohistochemical analysis for ICAM-1 was performed in the palm of hind paw sections as described in previous studies (Impellizzeri et al., 2016b). Sections were incubated overnight with anti-ICAM1 (1:100). Moreover, immunohistochemical analysis for COX-2 was performed in the spinal cord tissue, sections were incubated overnight with anti-COX2 (1:100). Controls included buffer alone or non-specific purified rabbit IgG. Sections were washed with PBS, incubated with secondary antibody. Specific labeling was detected with a biotin conjugated goat anti-rabbit IgG and avidin–biotin peroxidase complex (Vector; D.B.A s.r.l, Milan, Italy). The counter stain was developed with diaminobenzidine (brown color) and nuclear fast red (red background). Positive staining (brown color) was found in the sections, indicating that positive immunoreaction. The photographs obtained (*n* = 5 photos from each sample collected from all animals in each experimental group) were assessed by densitometry by using Leica QWin V3 United Kingdom). The percentage area of immunoreactivity was expressed as percent of total tissue area.

### Staining of Mast Cells

Mast cells identification was assessed in the palm of paw sections. Briefly, for evaluation of number of mast cells, tissue sections were stained with toluidine blue. Sections were deparaffinized in xylene and dehydrated through a graded series of ethanol, 5 min in each solution. The sections were next placed in water for 5 min, transferred to toluidine blue for 4 min and then blotted carefully. Sections were placed in absolute alcohol for 1 min, cleared in xylene, and mounted on a glass slide using Eukitt (Bio-Optica, Milan, Italy). Sections were stained blue and the mast cells were stained purple. Metachromatically stained mast cells were enumerated by counting five high-power fields (40×) per section using Axiovision Zeiss (Milan, Italy) microscope.

### Materials

Unless otherwise stated, all compounds used in this study were purchased from Sigma-Aldrich Company Ltd. (Poole, Dorset, United Kingdom). [^3^H]-PEA was purchased from Hartmann Analytic GmbH (Germany). [^14^C]-AEA was purchased from ARC (St. Louis, MO, United States). Unlabelled AEA was purchased from Tocris Bioscience (Avonmouth, Bristol, United Kingdom). Ultramicronized PEA and PEA-OXA were obtained from Epitech Group SpA, (Saccolongo, Italy). The immortalized HEK-293 cells over-expressing HEK-NAAA were obtained as reported previously (Saturnino et al., 2010). HEK-293 wild-type (HEK-WT) cells were purchased from LGC Standards (Milano, Italy). Deuterated standards - [^2^H]_4_-PEA, [^2^H]_8_-AEA, [^2^H]_5_-2-AG and [^2^H]_2_-OEA - were purchased from Cayman Chemical (Cabru, Arcore, Italy). All solutions used for *in vivo* infusions were prepared using non-pyrogenic saline (0.9% wt/vol NaCl; Baxter Healthcare Ltd., Thetford, Norfolk, United Kingdom).

### Statistical Evaluation

All values in the figures and text are expressed as mean ± standard deviation (SD), of N observations. For the experiment performed on HEK-NAAA cells, the figure shown is representative of at least three experiments. For *in vivo* studies N represents the number of animals studied. In the experiments involving histology, the figures shown are representative of at least three experiments performed on different days. The results were analyzed by one-way ANOVA followed by a Bonferroni *post hoc* test for multiple comparisons. The results of AEA, 2-AG, PEA and OEA levels were analyzed using the Student’s *t*-test. A *p*-value of less than 0.05 was considered significant.

## Results

### Effect of PEA-OXA on NAAA and FAAH Activity

**FIGURE 1:**
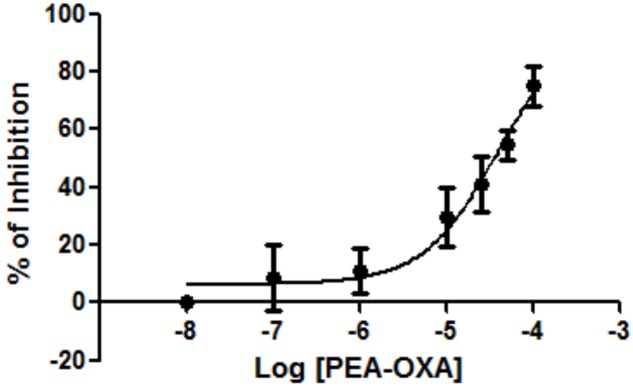
Effect of PEA-OXA on NAAA from HEK-293 cells overexpressing the human recombinant enzyme. Concentration-dependent inhibition of human recombinant NAAA by PEA-OXA was assessed. Mean values ± SD are shown.

In our experimental conditions, we found that PEA-OXA was able to inhibit NAAA by 75.0% at the maximal concentration tested (50 μM). The inhibition was concentration-dependent and did not reach a plateau, thus preventing the calculation of the IC_50_ (higher concentrations of PEA-OXA could not be used because unsoluble) (**Figure 1**). Pre-incubation of PEA-OXA for 20 min before the addition of substrate ([^3^H]-PEA) did not increase its inhibitory activity on NAAA. In fact, under these conditions, PEA-OXA exhibited only a 49% inhibition at the maximal concentration tested (data not shown), suggesting that PEA-OXA may also be a substrate of NAAA or of other enzymes present in the HEK293 cell membrane preparation used for the NAAA assay. However, PEA-OXA did not compete with AEA for hydrolysis by rat brain FAAH (maximal inhibition at 50 μM was 3%), an enzyme that is weakly expressed in HEK293 cells (van der Stelt et al., 2005) and can recognize as substrates not only PEA, but also OEA, AEA and 2-AG

### Effect of PEA-OXA on Time-Course of CAR-Induced Paw Edema in Rat

**FIGURE 2:**
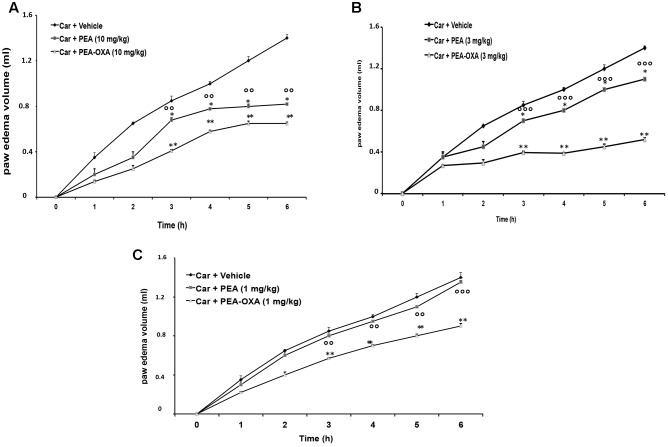
Effects of PEA-OXA at different doses on paw edema volume following CAR-injection in rats. Paw edema volume was assessed at the time points indicated **(A–C)** and with different doses of PEA-OXA or ultramicronized PEA (10, 3, and 1 mg/Kg; **A–C**, respectively). PEA-OXA, compared to PEA, produced significant reduction of paw volume in a dose dependent manner **(A–C)**. Values are shown as mean ± SD of 10 animals for each group. ^∗^*P* < 0.05 and ^∗∗^*P* < 0.01 vs. CAR; ^∘∘^*P* < 0.01 vs. PEA; ^∘∘∘^*P* < 0.001 vs. PEA.

Injection of CAR into the sub-plantar region of the left hind-paw rapidly induced a clear and time-dependent increase in paw edema volume, until 6 h (**Figures 2A–C**). A significant reduction of paw edema volume was observed in rats treated with 3 and 10 mg/kg ultramicronized PEA compared to the vehicle group (**Figures 2A,B**). Ultramicronized PEA at 1 mg/kg was not efficacious (**Figure 2C**). Moreover, treatment with PEA-OXA (1, 3 and 10 mg/kg) reduced significantly paw edema volume, showing a greater anti-inflammatory effect compared to the analogous non-cyclic PEA (**Figures 2A–C**).

### Effect of PEA-OXA on Levels of PEA, 2-AG, AEA and OEA in Rat Inflamed Plantar Paws

**FIGURE 3:**
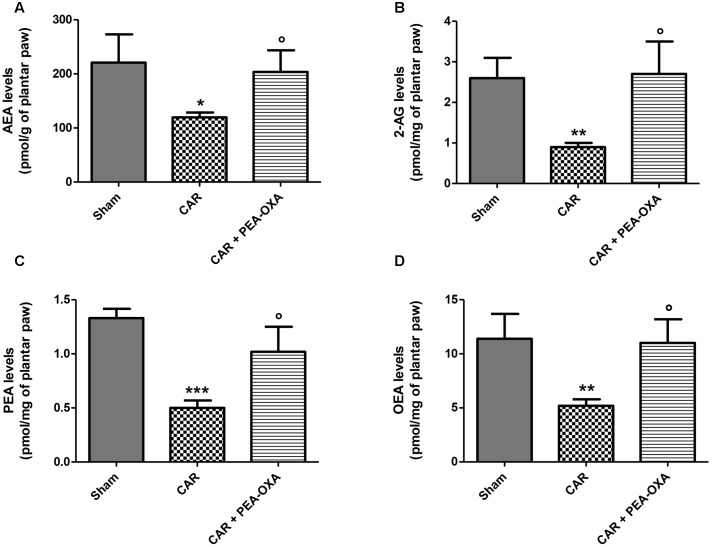
Effect of PEA-OXA on the levels of AEA, 2-AG, PEA and OEA in rat inflamed plantar paws. Endogenous levels of AEA **(A)**, 2-AG **(B)**, PEA **(C)**, and OEA **(D)** in paws of rats after intraplantar injection of CAR and treatment with PEA-OXA (10 mg/kg). Data are means ± SD of: *N* = 8 separate determinations for Sham group, *N* = 9 separate determinations for CAR group, *N* = 10 separate determinations for CAR+PEA-OXA. ^∗^*P* < 0.05 for Sham vs. CAR; ^∗∗^*P* < 0.01 for Sham vs. CAR; ^∗∗∗^*P* < 0.001 for Sham vs. CAR; °*P* < 0.05 for CAR vs. CAR+PEA-OXA.

In order to evaluate if the observed PEA-OXA anti-inflammatory effect is associated with modulation of FAAH and/or NAAA, the endogenous levels of AEA, 2-AG, PEA and OEA were evaluated in the inflamed plantar paws 6 h after CAR administration. As shown in **Figure 3**, a significant decrease of the endogenous levels of AEA, 2-AG, PEA, and OEA (**Figures 3A–D**, respectively) was observed in the plantar paws after CAR treatment. PEA-OXA treatment at 10 mg/kg restored the levels of the endogenous lipids to normal values (**Figures 3A–D**, respectively).

### Effect of PEA-OXA on Time-Course of CAR-Induced Thermal Hyperalgesia in Rat

**FIGURE 4:**
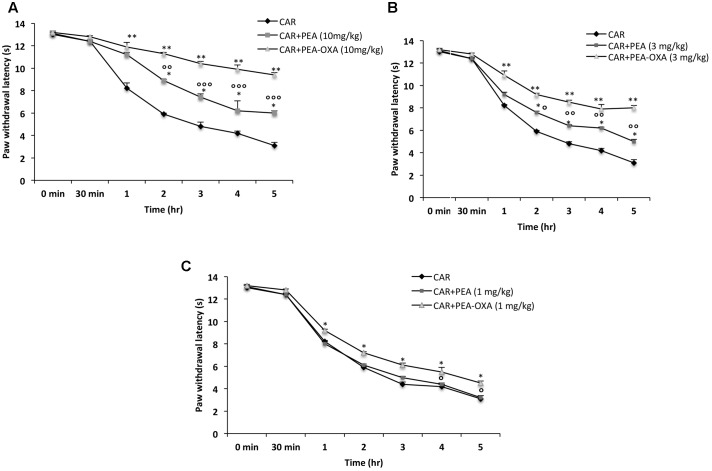
Effects of PEA-OXA at different doses on the time-course of CAR-induced thermal hyperalgesia in rats. Hyperalgesia were assessed at the time points indicated **(A–C)** with different doses of PEA-OXA or ultramicronized PEA (10, 3, and 1 mg/Kg; **A–C**, respectively). PEA-OXA, especially at the dose of 10 mg/kg, increased significantly paw latency in comparison to ultramicronized PEA. Values are shown as mean ± SD of 10 animals for each group. ^∗∗^*P* < 0.01 vs. CAR; ^∗^*P* < 0.05 vs. CAR; °*P* < 0.05 vs. PEA; ^∘∘^*P* < 0.01 vs. PEA; ^∘∘∘^*P* < 0.001 vs. PEA.

Intraplantar injection of CAR led to progress of thermal hyperalgesia maintained until 5 h (**Figures 4A–C**). Oral administration of ultramicronized PEA (3 and 10 mg/kg) produced a well-defined and significant inhibition in the development of CAR-induced thermal hyperalgesia compared to vehicle (**Figures 4A,B**); ultramicronized PEA at 1 mg/kg was not effective to reduce thermal hyperalgesia (**Figure 4C**). However, oral treatment with PEA-OXA (1, 3 and 10 mg/kg) was more significant than ultramicronized PEA in attenuating the CAR-induced hyperalgesic response (**Figures 4A–C**). These data confirmed the results obtained in a previous work (Impellizzeri et al., 2016a).

### Histological Analyses of Paw Tissues in CAR-Treated Rats

**FIGURE 5:**
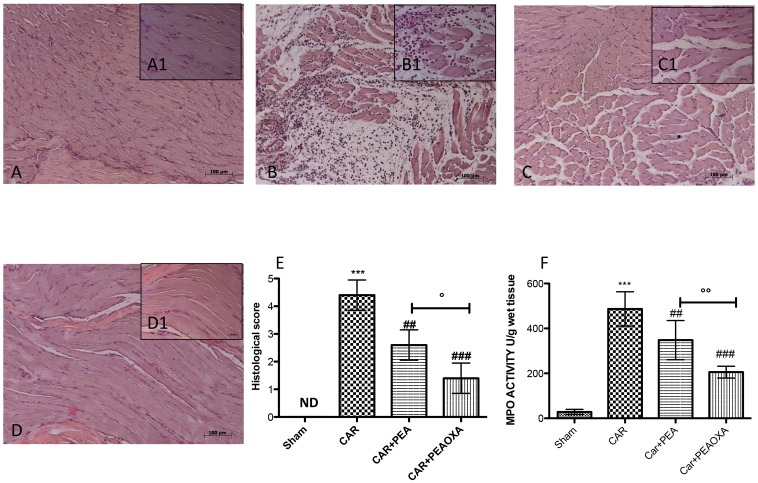
Anti-inflammatory effects of orally administered PEA-OXA following intraplantar injection of CAR in the rat hind paw: histological and biochemical analyses of MPO activity. Histological evaluation was performed by hematoxylin and eosin staining. Control **(A)** Intraplantar injection of CAR into the rat hind paw **(B)**, intraplantar injection of CAR with ultramicronized PEA (10 mg/kg; **C**); PEA-OXA (10 mg/kg; **D**). Insets **(A1)** through **(D1)** are higher-resolution (40X) images of the respective panels. Histological scores for the various treatment groups. **(E)** Myeloperoxidase (MPO) activity in paw tissues from the various treatment groups **(F)**. PEA-OXA produced significant improvements in both measurements compared to ultramicronized PEA. The histological score **(E)** was made by an independent observer according to this:*0 = no inflammation, 1 = mild inflammation, 2 = mild/moderate inflammation, 3 = moderate inflammation, 4 = moderate/severe inflammation, and 5 = severe inflammation.* The figure is representative of at least three experiments performed on different experimental days. Values are expressed as mean ± SD of 10 animals for each group. ^∗∗∗^*P* < 0.001 vs. sham; ^###^*P* < 0.001 vs. CAR; ^##^*P* < 0.01 vs. CAR; °*P* < 0.005 vs. PEA; ^∘∘^*P* < 0.01 vs. PEA.

Histological evaluation was made by H&E staining as described above. No histological damage was observed in control rats (**Figures 5A,A1**; see histological score **5E**), whereas important damage was observed 6 h after CAR injection with a marked accumulation of infiltrating inflammatory cells, edema and loss of normal muscle paw architecture (**Figures 5B,B1**; see histological score **5E**), compared to control. Histological damage was significantly decreased upon treatment with ultramicronized PEA (**Figures 5C,C1** and see histological score **5E**). However, PEA-OXA (**Figures 5D,D1** and histological score **5E**) reduced morphological alterations to a greater extent than ultramicronized PEA. Histological damage was associated with an increased neutrophil infiltration as shown by intensification in MPO activity (**Figure 5F**). Administration of ultramicronized PEA (10 mg/kg) reduced MPO activity (**Figure 5F**), and PEA-OXA (10 mg/kg) was more significant than PEA in this respect (**Figure 5F**).

### Effect of PEAOXA on Mast Cell Number in Paw Tissue from CAR-Treated Rats

**FIGURE 6:**
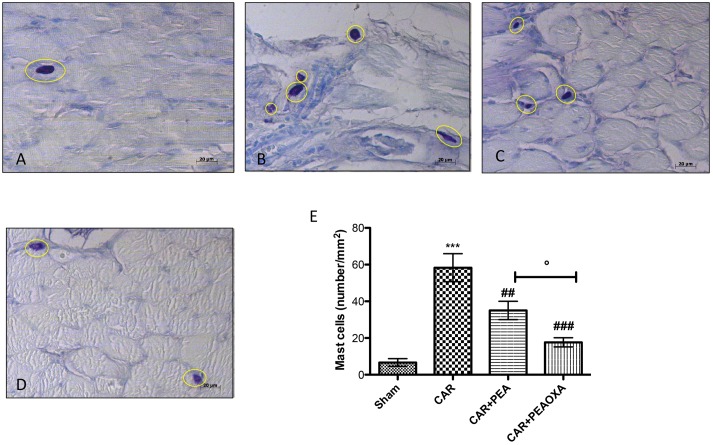
Staining for mast cell number in rats. The mast cells were identified in the slides stained with acidified toluidine blue that have dark lilac blue granules. Panels **(A)**, control group; Panels **(B)**, CAR group; Panels **(C)**, CAR+PEA group; Panels **(D)**, CAR+PEA-OXA group. The number of mast cells per unit area of tissue (mast cell density) is shown in the graph **(E)**. PEA-OXA (10 mg/kg) was able to reduce the number of mast cells more efficaciously than ultramicronized PEA (10 mg/kg). Metachromatically stained mast cells were enumerated by counting five high-power fields (40×) per section using Axiovision Zeiss (Milan, Italy) microscope. Values are expressed as mean ± SD of 10 animals for each group. ^∗∗∗^*P* < 0.001 vs. sham; ^###^*P* < 0.001 vs. CAR; ^##^*P* < 0.01 vs. CAR; °*P* < 0.005 vs. PEA.

Mast cells and histamine play a key role in edematogenic activity (Kimura et al., 2015). Using toluidine blue staining to evidence mast cells, we detected a presence of mast cells in paw tissues 6 h after edema induction (**Figure 6B**; see mast cell count **6E**) compared to sham animals (**Figure 6A**; see mast cell count **6E**). In contrast, a lower number of mast cells was found in paw tissues from CAR mice treated with PEA-OXA (**Figure 6D**; see mast cell count **6E**) compared to the PEA group (**Figure 6C**; mast cell count **6E**).

### Effect of PEA-OXA on ICAM-1 Expression in Paw Tissue from CAR-Treated Rats

**FIGURE 7:**
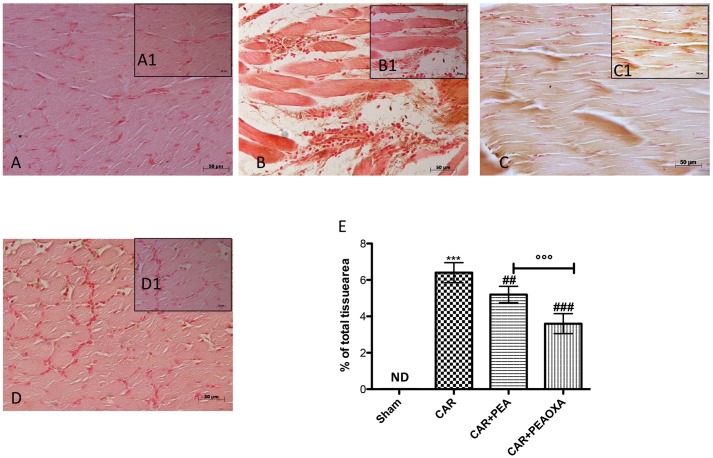
Immunohistochemical analysis of ICAM in the rat hind paw tissues following intraplantar hind paw injection of CAR and PEA-OXA. ICAM expression was evaluated by immunohistochemical analysis. Panels **(A,A1)**, control group; Panels **(B,B1)**, CAR group; Panels **(C,C1)**, CAR+ ultramicronized PEA (10 mg/kg) group; Panels **(D,D1)**, CAR+PEA-OXA (10 mg/kg) group. Densitometry analysis of immunocytochemistry photographs (*n* = 5) for ICAM from paw sections was assessed **(E)**. The percentage of positive immunostaining (% brown staining) as a function of total tissue area was quantified. Values are expressed as mean ± SD of 10 animals for each group. ^∗∗∗^*P* < 0.001 vs. sham; ^###^*P* < 0.001 vs. CAR; ^##^*P* < 0.01 vs. CAR; ^∘∘∘^*P* < 0.001 vs. PEA.

Immunohistochemical analysis for ICAM-1 showed constitutive expression of this adhesion molecule in paw tissue sections from saline-treated rats (**Figures 7A,A1**; see densitometric analysis **7E**). Six hours after CAR injection saw a substantial increase in ICAM-1 staining intensity (brown staining) along the paw tissue (**Figures 7B,B1**; see densitometric analysis **7E**), which was significantly attenuated in paw tissue of CAR-treated rats that received PEA-OXA (10 mg/kg) (**Figures 7D,D1**; see densitometric analysis **7E**) compared to the ultramicronized PEA group (**Figures 7C,C1**; see densitometric analysis **7E**).

### Effects of PEA-OXA on Cytokine Release from Paw Tissue of CAR-Treated Rats

**FIGURE 8:**
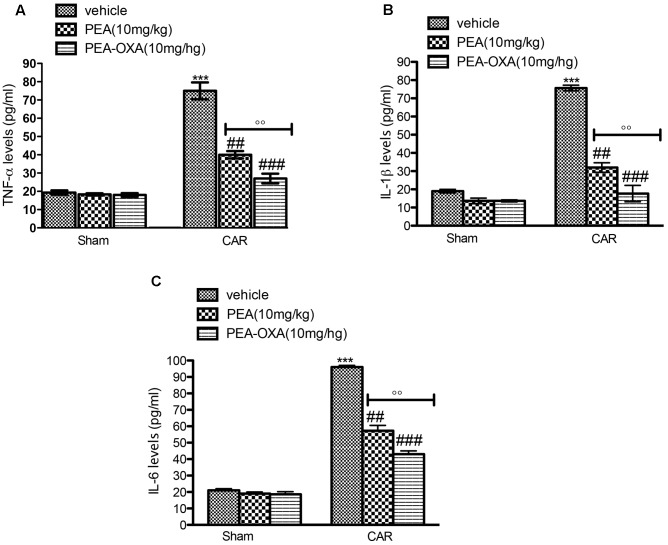
ELISA assay of TNF-α, IL-1β, IL-6 in the rat hind paw tissues following intraplantar hind paw injection of CAR: effects of administered PEA-OXA. An increase of TNF-α **(A)**, IL-1β **(B)**, IL-6 **(C)**, levels were found in CAR+vehicle rats after CAR induction. CAR-injected rats and treated with ultramicronized PEA (10 mg/kg) exhibited a reduction of TNF-α **(A)**, IL-1β **(B)**, IL-6 **(C)** levels. PEA-OXA treatment (10 mg/kg) reduced markedly cytokine expression **(A–C)**. Values are shown as mean ± SD of 10 animals for each group. ^∗∗∗^*P* < 0.001 vs. sham; ^###^*P* < 0.001 vs. CAR; ^##^*P* < 0.01 vs. CAR; ^∘∘^*P* < 0.01 vs. PEA.

Inhibition of edema and hyperalgesia was associated with inhibition of pro-inflammatory and pro-nociceptive cytokines, such as TNF-α, IL-1β, and IL-6. Administration of ultramicronized PEA (10 mg/kg) partially decreased cytokine release in paw tissues (**Figures 8A–C**, respectively). Moreover, PEA-OXA (10 mg/kg) induced an important reduction of cytokines expression (**Figures 8A–C**, respectively). TNF-α, IL-1β, and IL-6 production was clearly evident in the CAR group (**Figures 8A–C**, respectively) compared to sham.

### Effect of PEA-OXA on Expression of IκB-α and Nuclear Translocation of NF-κB p65 in Paw Tissue of CAR-Treated Rats

**FIGURE 9:**
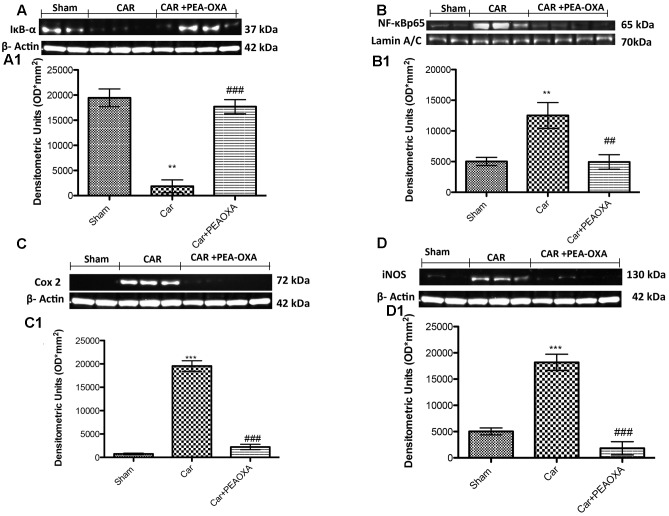
Western blot analysis of IκB-α, NF-κB p-65, COX-2, and iNOS in the rat hind paw tissues following intraplantar hind paw injection of CAR: effects of administered PEA-OXA. The basal level of IκB-α in paw tissue homogenates from control rats **(A,A1)** decreased after CAR injection, and treatment with PEA-OXA (10 mg/kg) decrease significantly IκB-α degradation. Moreover, NF-κB p-65 levels in paw **(B,B1)** tissues were significantly increased after CAR induction; PEA-OXA (10 mg/kg) markedly reduced NF-κB p-65 translocation **(B,B1)**. CAR-injected rats also showed an increase in COX-2 and iNOS expression (**C,C1,D,D1**, respectively), PEA-OXA (10 mg/kg) significantly reduced COX-2 and iNOS expression (**C,C1,D,D1**, respectively). Data are representative of at least three independent experiments. Values are means ± SD of 10 animals for each group. ^∗∗∗^*P* < 0.001 vs. sham; ^∗∗^*P* < 0.01 vs. sham; ^###^*P* < 0.001 vs. CAR; ^##^*P* < 0.01 vs. CAR.

To better understand the mechanism of action of PEA-OXA, we also investigated the effects on the NF-κB pathway. Basal expression of IκB-α was detected in paw tissues from control animals (**Figure 9A**, see densitometric analysis **9A1**), whereas IκB-α degradation was substantially increased in paw tissues from CAR-injected rats (**Figure 9A**, see densitometric analysis **9A1**). Treatment with PEA-OXA (10mg/kg) significantly prevented CAR-induced IκB-α degradation, in terms of increased IκB-α levels (**Figure 9A**, see densitometric analysis **9A1**). Moreover, p65 subunit translocation was increased after CAR injection in paw tissue compared to sham (**Figure 9B**, see densitometric analysis **9B1**). Treatment with PEA-OXA (10mg/kg) reduced significantly p65 translocation (**Figure 9B**, see densitometric analysis **9B1**).

### Effect of PEA-OXA on Expression of iNOS and COX-2 in Paw Tissue of CAR-Treated Rats

We also determined the effect of PEA-OXA (10 mg/kg) on pro-inflammatory enzymes such as COX-2 and iNOS after CAR injection. The expression of COX-2 was increased in paw tissues after CAR injection compared to control rats (**Figure 9C**; see densitometry analysis **9C1**). On the other hand, rats treated with PEA-OXA show a great reduction in COX-2 (**Figure 9C**; see densitometry analysis **9C1**). iNOS was also assessed by WB analysis in paw homogenates 6 h after CAR-induced paw edema (**Figures 9D,D1**). There was a significant increase of iNOS expression in the CAR group (**Figure 9D**, see densitometric analysis **9D1**). Treatment with PEA-OXA significantly reduced iNOS expression in paw tissues (**Figure 9D**, see densitometric analysis **9D1**).

### Analgesic Effects of PEA OXA in Lumbar Spinal Cord Tissues after CAR Injection in Rats

**FIGURE 10:**
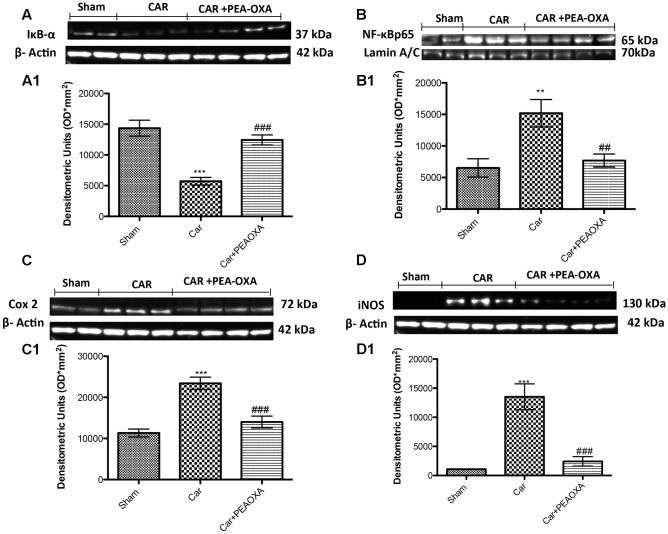
Western blot analysis of IκB-α, NF-κB p-65, COX-2 and iNOS in the rat spinal cord tissues following intraplantar hind paw injection of CAR: effects of administered PEA-OXA. Control group in spinal cord tissue homogenates showed a constitutive level of IκB-α **(A,A1)** that decreased after CAR injection. Treatment with PEA-OXA (10 mg/kg) decreases significantly IκB-α degradation **(A,A1)**. Likewise, translocation in the nucleus of NF-κB p-65 in spinal cord **(B,B1)** tissues was considerably increased after CAR induction; PEA-OXA (10 mg/kg) markedly reduced NF-κB p-65 translocation. CAR-injected rats also showed an increase in COX-2 and iNOS expression (**C,C1,D,D1**, respectively) in the spinal cord tissue, PEA-OXA (10 mg/kg) significantly reduced COX-2 and iNOS expression (**C,C1,D,D1**, respectively). Data are representative of at least three independent experiments. Values are means ± SD of 10 animals for each group. ^∗∗∗^*P* < 0.001 vs. sham; ^∗∗^*P* < 0.01 vs. sham; ^###^*P* < 0.001 vs. CAR; ^##^*P* < 0.01 vs. CAR.

**FIGURE 11:**
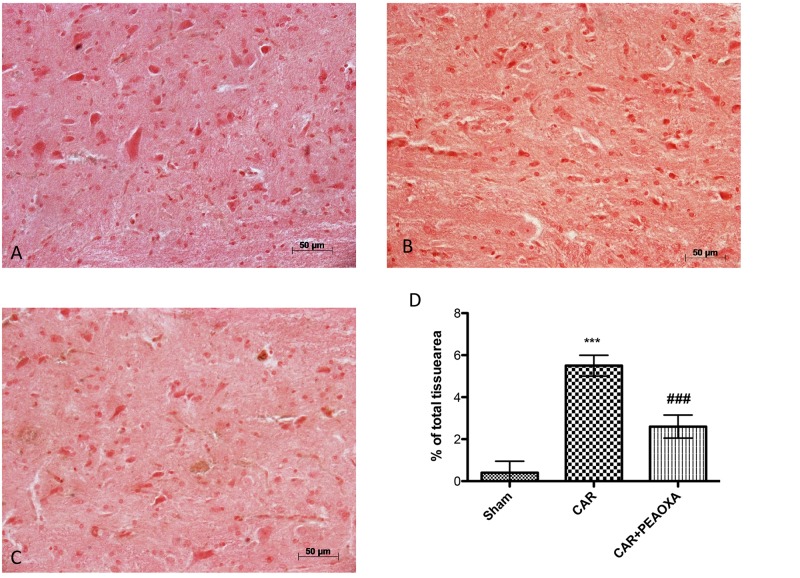
Anti-inflammatory effects of PEA-OXA on COX-2 expression. COX-2 expression was also evaluated by immunohistochemical analysis. Panels **(A)** represent control group; Panels **(B)** represent CAR group; Panels **(C)** represent CAR+ PEA-OXA (10 mg/kg) group; Densitometry analysis of immunocytochemistry photographs (*n* = 5) for ICAM from paw sections was assessed **(D)**. The percentage of positive immunostaining (% brown staining) as a function of total tissue area was quantified. Values are expressed as mean ± SD of 10 animals for each group. ^∗∗∗^*P* < 0.001 vs. sham; ^###^*P* < 0.001 vs. CAR.

We next assessed whether the analgesic effects of PEA-OXA were related to spinal inhibition of inflammatory stress through modulation of the NF-κB pathway and inflammatory proteins. Intraplantar injection of CAR was associated with a prominent degradation of IKB-α and reduction of NF-κBp65 nuclear translocation, as well as a marked increase in COX-2 and iNOS expression in lumbar spinal cord tissues (**Figures 10A–D**, respectively; see densitometric analysis **10A1–D1**). PEA-OXA (10mg/kg) significantly reduced IKB-α degradation (**Figure 10A**) and the expression of NF-κB, iNOS and COX-2 (**Figures 10B–D**) in spinal cord tissue. Moreover, by immunohistochemistry analysis we confirm an increasing staining intensity for COX-2 after CAR injection (**Figure 11B** see densitometric analysis **11D**); compared to the sham group (**Figure 11A**), whereas treatment with PEA-OXA significantly attenuated the expression of COX-2 decreasing staining intensity of COX-2 as show in **Figure 11C**, see densitometric analysis **11D**.

### Effects of PEA OXA on PPAR- α after CAR Injection in Mice

**FIGURE 12:**
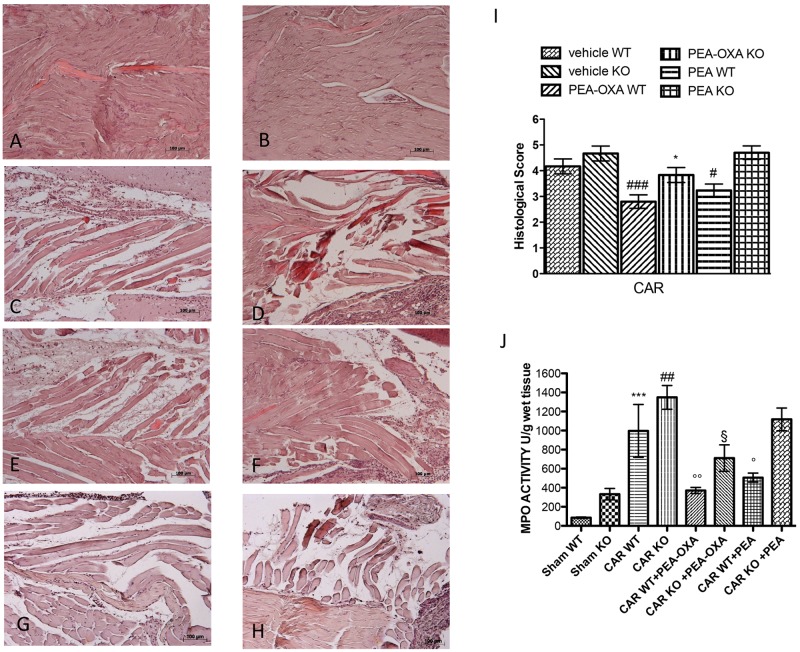
Anti-inflammatory effects of orally administered PEA-OXA following intraplantar injection of CAR in the hind paw in PPAR-α WT and KO mice: histological and biochemical analyses of MPO. Hematoxylin and eosin staining performed histological evaluation. Sham WT and PPAR-α KO (**A,B**, respectively); intraplantar injection of CAR in WT and PPAR-α KO (**C,D**, respectively); intraplantar injection of CAR with PEA-OXA (10 mg/kg) in WT and PPAR-α KO (**E,F**, respectively). Ultramicronized PEA was able to reduce in a significant way the histological damage in WT mice **(G)** but not in PPAR-α KO mice **(H)**, whereas PEA-OXA was able to reduce significantly paw tissue modification in WT mice **(E)** and in KO mice **(F)**. Histological scores for the various treatment groups **(I)**. Measure of MPO activity after CAR injection **(J)**. Ultramicronized PEA was able to reduce in a significant manner MPO activity in WT but not in PPAR-α KO mice **(H)**; PEA-OXA reduced significantly MPO activity in both PPAR-α KO and WT mice **(H)**. For histological score data are representative of at least three independent experiments. Values are means ± SD of 10 animals for each group: ^###^*P* < 0.001 vs. vehicle WT; #*P* < 0.05 vs. vehicle WT; ^∗^*P* < 0.05 vs. vehicle KO. For MPO data are representative of at least three independent experiments. Values are means ± SD of 10 animals for each group: ^∗∗∗^*P* < 0.001 vs. Sham WT; ^##^*P* < 0.01 vs. Sham KO; ^∘∘^*P* < 0.01 vs. CAR WT; °*P* < 0.05 vs. CAR WT; ^§^ *P* < 0.05 vs. CAR KO.

To interrogate a role for PPAR-α activation in the mechanism of action of PEA-OXA, we repeated these experiments in PPAR-α KO mice. CAR injection in the hind paw resulted in severe histological alterations in the tissue architecture in paws from PPAR-α KO mice compared to WT mice (**Figures 12C,D**, respectively, see histological score **12G**). Ultramicronized PEA treatment (10mg/kg) reduced this histological damage in WT mice but failed to do so in PPAR-α KO mice (**Figures 12G,H**, respectively, see histological score **12G**). On the contrary, PEA-OXA (10mg/kg) reduced significantly paw tissue histopathology in both WT and PPAR-α KO mice (**Figures 12E,F**, respectively, see histological score **12G**) compared to ultramicronized PEA (10mg/kg) (**Figures 12G,H**, respectively, see histological score **Figure 11G**). No histological alterations were seen in sham mice (**Figures 12A,B**, see histological analysis **12G**). Further, ultramicronized PEA treatment (10mg/kg) decreased neutrophil infiltration (MPO activity) in WT but not in PPAR-α KO mice (**Figure 12J**). In contrast, PEA-OXA (10mg/kg) reduced markedly MPO activity in PPAR-α KO and WT mice more than ultramicronized PEA (**Figure 12J**) suggesting a possible alternative mechanism of action for this molecule.

### Effects of PEA-OXA on Time-Course of CAR-Induced Paw Edema Volume in PPAR-α KO Mice

**FIGURE 13:**
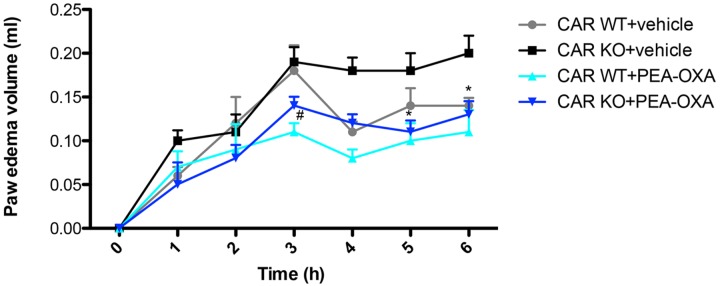
Effects of PEA-OXA on the time course of CAR-induced paw edema volume following intraplantar injection of CAR in the hind paw in PPAR-α WT and KO mice. Paw edema volume was assessed at the time points indicated. PEA-OXA treatment (10 mg/kg) significantly decreased paw edema volume in PPAR-α KO and WT groups. Data are representative of at least three independent experiments. Values are means ± SD of 10 animals for each group ^∗^*P* < 0.05 vs. CAR KO; ^#^*P* < 0.05 vs. CARWT.

Intraplantar injection of CAR caused a time-dependent paw edema volume increase in PPAR-α KO and WT mice (**Figure 13**). Oral administration of PEA-OXA (10 mg/kg) markedly reduced paw edema volume in CAR-injected WT mice. However, PEA-OXA treatment (10 mg/kg) also significantly decreased paw edema volume in CAR-injected PPAR-α KO mice (**Figure 13**).

## Discussion

Inflammation, a physiological self-defense mechanism in response to systemic or local stimuli (Myers et al., 2006), is generally classified as acute or chronic. CAR-induced paw edema and hyperalgesia is a helpful model to evaluate the contribution of mediators in vascular changes associated with acute inflammation. The development of edema has been considered as a complex event in which various mediators produce the inflammatory response (Salvemini et al., 1996) that characterizes a great many diseases. The present study was designed to investigate novel pharmacological modalities for modulating the inflammatory process involved in the development of paw edema, at both peripheral and central levels, through suppression of specific inflammatory mediators considered crucial for inflammatory disease. In particular, we used a new compound PEA-OXA that displays protective and anti-nociceptive effects, by markedly reducing the inflammation-associated paw edema, with overall inhibition of the effects of reactive oxygen species exerted both peripherally and centrally.

We previously showed that PEA regulates nociception and inflammation in a model of CAR-induced paw edema (Impellizzeri et al., 2014), resulting in a marked inhibition of edema, cytokine production and reactive oxygen species formation. PEA, following its on-demand production by the organism in response to specific stimuli, is degraded by NAAA, a lysosomal cysteine amidase. In previous studies, the inhibition of NAAA led to increased tissue levels of PEA and reduced hyperalgesic responses and pro-inflammatory cytokine production (Bandiera et al., 2014; Impellizzeri et al., 2016a; Migliore et al., 2016). Based on these previous studies, we utilized a new compound, PEA-OXA, and demonstrated that it is capable to inhibit the activity of NAAA and thus increase the tissue levels of PEA (that were reduced by CAR intraplantar injection in the right hindpaw). Accordingly, oral treatment with PEA-OXA reduced tissue damage, thermal hyperalgesia, and accumulation of infiltrating inflammatory cells in the hind paw (MPO activity), while proving to be more efficacious than ultramicronized PEA at the same dose. The inhibitory activity of PEA-OXA on NAAA was assessed *in vitro* using the human recombinant, and not the rat, enzyme.

Early stages of inflammation generally result in up-regulation of activated transcription factors such as NF-κB, which is known to induce the expression of pro-inflammatory enzymes such as COX-2 and iNOS, which lead to biosynthesis of prostanoids and NO as well as the release of pro-inflammatory cytokines such as TNF-α (D’Agostino et al., 2007). NF-kB is sequestered in the cytoplasm by IκB in a quiescent state; upon its activation, IκB is phosphorylated by the IκB kinase complex leading to its degradation and to the nuclear translocation of NF-κB, which in turn initiates the downstream transcription of target pro-inflammatory genes (Liu et al., 2012). Here, orally administered PEA-OXA reduced degradation of IκB-α as well as the nuclear translocation of NF-κB. As a consequence of this latter action, PEA-OXA decreased the levels of inflammatory enzymes under NF-κB control (COX-2, iNOS) in spinal cord and paw tissues and, subsequently, of their products, being more efficacious than ultramicronized PEA given orally at the same dose.

Peripheral inflammation involves an increase in COX-2-mediated prostaglandin synthesis in the CNS, which contributes to nociception and hyperalgesia and determines the second accelerating phase of swelling (1–6 h) in paw edema (Maihofner et al., 2000). Likewise, peripheral damage causes an increase in COX-2 and iNOS expression, which are involved in inflammatory signaling to the CNS. In fact, COX-2 is rapidly induced in the spinal cord and other CNS regions following CAR injection in the hind paw (Ichitani et al., 1997), where it plays a pivotal role in sustaining pain and peripheral inflammation as we already demonstrated in our previous study (Esposito et al., 2016).

In this regard, we propose that PEA-OXA, by blocking NF-κB at the peripheral level, leads to a reduced production of iNOS and COX-2 centrally. Thus, the strong reduction of iNOS and COX-2 mediated by PEA-OXA produced in the periphery is sufficient to significantly prevent downstream effectors associated with inflammatory pain in the spinal cord. Here we demonstrated that after CAR injection there is an increased expressions of both iNOS and COX2 at spinal cord levels, expressions that were significantly attenuated by PEA-OXA treatment.

Mast cells are multifunctional immune cells that contain a variety of inflammatory mediators. The various cytokines and several bioactive substances, e.g., neuropeptides, and the kinins, which are supplied from tissue microenvironments, are believed to act as positive or negative regulators of mast cell function (Mortaz et al., 2005). The early inflammatory response of CAR-induced edema in rats results from the release of histamine and serotonin from mast cells (Carvalho et al., 2006), and mast cell numbers have been reported to be increased in inflammatory conditions (Wang et al., 2009). Moreover, has been reported that during inflammation the increased expression of pro-inflammatory cytokines is dependent by the activation of the transcription factor NF-κB (Baeuerle and Baltimore, 1988). In this study, we showed that PEA-OXA is able to reduce mast cell number and pro-inflammatory cytokine levels during paw inflammation via the inhibition of NF-κB activation.

To investigate the mechanism by which PEA-OXA attenuated the development of CAR-induced paw edema, we asked whether PPAR-α could have a role in mediating the anti-inflammatory and anti-nociceptive effects of PEA-OXA. When CAR-induced paw edema was performed in PPAR-α KO mice, the protective effects of PEA-OXA were unaltered, unlike those of ultramicronized PEA, the effects of which were completely dependent on PPAR-α activation. Thus, the effects of PEA-OXA appear to operate via a molecular pathway independent of PPAR-α.

Accordingly, although PEA-OXA administration resulted in the elevation of PEA levels in the paw of CAR-treated-rats (which, as shown previously (Costa et al., 2010; Petrosino et al., 2016) contain lower levels of this and other related mediators), it also elevated the levels of the endocannabinoids AEA and 2-AG, and those of the PEA congener, OEA. These mediators could also lead to the activation of their molecular targets, which do not necessarily include PPAR-α. Whilst OEA and AEA are also (poor) substrates for NAAA, 2-AG is not. Therefore, the effects of PEA-OXA on the levels of these other mediators might be the mere consequence of the amelioration of edema and, hence, of the reduction of tissue wet weight (with subsequent increase of all mediator concentrations). It is also possible that PEA-OXA might act via a more efficacious “entourage” effect on the levels of OEA, PEA and 2-AG than exogenous PEA, which has never been found to elevate the levels of all three other compounds simultaneously via this effect (Petrosino and Di Marzo, 2016). It is unlikely that such effects are due to direct inhibition of FAAH, since PEA-OXA does not inhibit AEA hydrolysis by the rat enzyme.

## Conclusion

We demonstrate that PEA-OXA, at least in part through the inhibition of the PEA-catabolizing enzyme NAAA, exerts anti-inflammatory and anti-nociceptive effects by suppressing the release of inflammation-associated mediators (TNF-α, IL-6, reactive oxygen species), reducing COX-2 and iNOS over-expression, and inhibiting NF-κB activation at spinal cord levels. These anti-inflammatory actions of PEA-OXA were stronger than those of the analogous non-cyclic fatty acid amide PEA.

These data should encourage the further testing of PEA-OXA as a potentially clinically relevant alternative or add-on to PEA, which is currently marketed against chronic pain.

## Author Contributions

IP and MCa prepared the manuscript. DI and EG performed the *in vivo* experiments. RD, RS, and MCo performed western blot analysis and immunohistochemical staining. MA performed the Enzyme preparation. SP performed LC-APCI-MS analysis. SP, VDM, SC, and EE planned experiments and analyzed the results. All authors read and reviewed the manuscript.

## Conflict of Interest Statement

SC and VDM, researchers on the study team, are co-inventor on patent WO2013121449 A8 (Epitech Group SpA) which deals with compositions and methods for the modulation of amidases capable of hydrolysing *N*-acylethanolamines useable in the therapy of inflammatory diseases. Moreover, SC is also a co-inventor with Epitech group on the following patents: (1) EP2821083, (2) EP 2985037 A1, (3) 102015000067344. SP and MA are employees of Epitech Group SpA. The other authors declare that the research was conducted in the absence of any commercial or financial relationships that could be construed as a potential conflict of interest.
